# Usefulness of Once-Weekly GLP-1 Receptor Agonist Semaglutide on Glycemic Control in Subjects with Type 2 Diabetes Mellitus: Switching from the Same Class Dulaglutide in a Retrospective Observation Study

**DOI:** 10.1155/2024/5880589

**Published:** 2024-01-04

**Authors:** Tomohiko Kimura, Masato Kubo, Kaio Takahashi, Ryo Wamata, Yuichiro Iwamoto, Hideyuki Iwamoto, Yukino Katakura, Junpei Sanada, Yoshiro Fushimi, Masashi Shimoda, Fuminori Tatsumi, Shuhei Nakanishi, Tomoatsu Mune, Kohei Kaku, Hideaki Kaneto

**Affiliations:** Department of Diabetes, Endocrinology and Metabolism, Kawasaki Medical School, Kurashiki, Japan

## Abstract

Recently, the development of once-weekly incretin-based injections dulaglutide and semaglutide has drawn a great deal of attention. This study is aimed at comparing the efficacy of once-weekly GLP-1 receptor activator (GLP-1RA) dulaglutide and semaglutide on glycemic control and several metabolic parameters in patients with type 2 diabetes mellitus. We compared various clinical parameters between before and after switching from dulaglutide to semaglutide in “study 1” (pre-post comparison) and set the control group using propensity score matching method in “study 2.” In “study 1,” six months after the switching, HbA1c was significantly reduced from 8.2% to 7.6% and body mass index was also decreased from 30.4 kg/m^2^ to 30.0 kg/m^2^. Such effects were more pronounced in subjects whose glycemic control was poor. In “study 2,” after 1 : 1 propensity score matching, glycemic control and body weight management were improved in the switching group compared with the dulaglutide continuation group. In this study including obese subjects with poor glycemic control, switching dulaglutide to semaglutide showed more beneficial effects on both glycemic and weight control irrespective of age, body weight, and diabetes duration. Therefore, we should bear in mind that it would be better to start using a relatively new once-weekly GLP-1RA semaglutide in clinical practice, especially in obese subjects with poor glycemic control with other GLP-1RAs.

## 1. Introduction

It is well known that type 2 diabetes mellitus (T2DM) causes not only microangiopathy but also macroangiopathy, especially in subjects with poor glycemic control. Some large clinical trials, such as DCCT, UKPDS, and Kumamoto studies, showed that meticulous glycemic management prevented the progression of diabetic microangiopathy [1–3]. However, ADVANCE and ACCORD studies, conducted in subsequent years, suggested that avoidance of hypoglycemia is very important in glycemic control [4, 5].

These days, incretin modulators are frequently used in clinical practice because of their usefulness. Because incretin modulators stimulate insulin secretion in response to blood glucose levels, hypoglycemia risk is pretty low with monotherapy of these drugs. There are two types of incretin modulators: dipeptidyl peptidase-4 (DPP-4) inhibitors and GLP-1 receptor agonists (GLP-1RA). It has been shown that major cardiovascular events can be prevented by several GLP-1RAs [6]. In addition, GLP-1RA can reduce body weight, mainly through the effect of appetite suppression [7]. Recently, it has been revealed that the use of GLP-1RA is associated with lower mortality [8]. At present, various types of GLP-1RAs are available. Some GLP-1RAs are injected once a week, including dulaglutide and semaglutide. When patients with T2DM do not have adequate glycemic control or weight management, switching among GLP-1RA classes can be considered [9–12]. We reported a retrospective study showing that switching from DPP-4 inhibitor to GLP-1RA dulaglutide improved glycemic control [13]. In Japan, once-weekly GLP-1RAs, dulaglutide (0.75 mg) and semaglutide (0.25 mg, 0.5 mg, and 1.0 mg) are available for the treatment of T2DM. There are several reports showing the difference in efficacy among several GLP-1RAs. For example, the SUSTAIN 7 trial directly compared the efficacy of semaglutide and dulaglutide [14]. As the result, semaglutide more significantly ameliorated glycemic control and reduced body weight compared to dulaglutide, especially when a high dose was administered. However, there are only a few reports showing the efficacy of switching from one GLP-1RA to another one on HbA1c value and weight control. For example, in REALISE-DM study, switching from liraglutide or dulaglutide to semaglutide decreased HbA1c value by 0.65% and body weight by 1.69 kg for 6 months [15]. However, the dose of dulaglutide was different from that approved in Japan.

**Figure 1:**
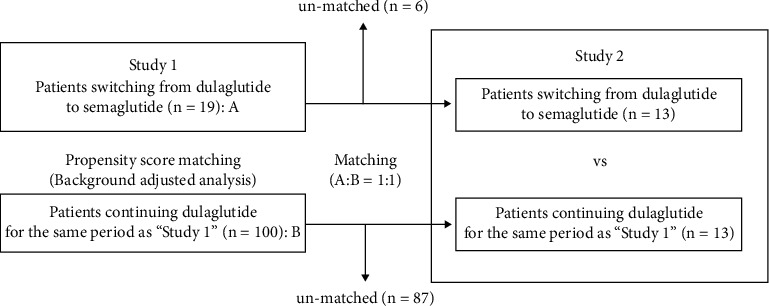
Study schema.

Therefore, in this study, we examined the efficacy of switching once-weekly GLP-1RA dulaglutide to semaglutide on HbA1c value, body weight, and various parameters in T2DM subjects (study 1). Since the absence of a control group is often considered as a problem in switching studies, patients who continued to receive dulaglutide during the same period were matched at a ratio of 1 : 1 using the propensity score (PS) matching method and used as controls (study 2) (Figure 1).

## 2. Methods

### 2.1. Study Population and Patient Preparation

This study was conducted retrospectively in outpatients of our hospital from June 1 in 2021 to December 28 in 2021. The research protocol including the opt-out informed consent was approved by the Institutional Review Board of Kawasaki Medical School (No. 5479). And this study was conducted according to the Declaration of Helsinki. Because this study was retrospective, public information about the study was provided through the hospital homepage instead of obtaining informed consent from each subject. Subjects enrolled in this study met the criteria as follows: (1) subjects with T2DM who was already taking dulaglutide, (2) without severe liver dysfunction, (3) without severe renal dysfunction, (4) without any malignant disease, (5) without any infectious or endocrine diseases, and (6) without receiving steroid therapy. Cases with changes in antidiabetic drugs during the observation period were not included.

### 2.2. Clinical Parameters

We measured clinical parameters, including height, BMI, glucose and lipid parameters, and liver and renal function. The primary outcome was a change of HbA1c during the 6 months after switching from dulaglutide to semaglutide “study 1” (pre-post comparison).

### 2.3. Switching and Dosing of Once-Weekly GLP-1RAs

The participants in this study were treated with dulaglutide (0.75 mg/week). At the time of switching, each attending physician decided the dose of semaglutide. Afterwards, the dose could be increased up to 1.0 mg at the discretion of the attending physician. Namely, the final dose of semaglutide was not predetermined and was judged by each attending physician according to each patient's condition. After switching once-weekly GLP-1RA dulaglutide to semaglutide, we evaluated HbA1c values, body weight, and other clinical parameters at the time of switching and six months later. A total of 19 subjects (male/female = 15/4) were enrolled, and we collected the clinical data in this study subjects. This study also used data from 3 months before the switching.

### 2.4. Statistical Analysis

JMP version 13 (SAS Institute Inc., North Carolina, USA) was used in all statistical analyses. A paired *t*-test was conducted to compare differences between time points in the same group. A Mann–Whitney *U* test was performed to compare the difference between the improved and nonimproved HbA1c groups. To determine which factors correlated with *Δ*HbA1c, we performed Spearman's rank correlation coefficient test. In study 2, we set the control group who were continuously treated with dulaglutide without changing any antidiabetic agents within the same period time. Next, to minimize imbalances in clinical parameters between each group at baseline, one versus one propensity score matching was performed with the switching (dulaglutide to semaglutide) group. Furthermore, in this method, we included five predefined baseline covariates: age, gender, diabetes duration, HbA1c, and BMI. *χ*^2^ test was used for comparing the usage rate of concomitant drugs between the 2 groups. In the above-mentioned analyses, *p* < 0.05 was considered significant. The results were expressed as mean ± SD.

## 3. Results

### 3.1. Clinical Features in This Study Subjects

#### 3.1.1. Study 1

We enrolled a total of 19 subjects (male/female = 15/4) (Table 1(a)). The baseline characteristics of the subjects in this study were as follows: age, 53.1 ± 11.1 years old; BMI, 30.4 ± 5.2 kg/m^2^; diabetes duration, 16.9 ± 10.4 years; HbA1c, 8.2 ± 1.1%; and plasma glucose (PG), 156.9 ± 59.7 mg/dL. Frequencies of subjects having diabetic neuropathy, retinopathy, and nephropathy (urinary albumin ≥ 30 mg/gCr) were 42.1%, 36.8%, and 52.6%, respectively. Frequencies of subjects suffering from ischemic heart disease and stroke were 15.8% and 10.5%, respectively. The percentages of using metformin, DPP-4 inhibitor, SGLT2 inhibitor, sulfonylurea, glinide, thiazolidine, *α*-glucosidase inhibitor, and insulin preparation were 84.2%, 0%, 73.7%, 31.6%, 5.2%, 52.6%, 10.5%, and 31.6%, respectively. Percentages of subjects taking some medicine for hypertension and dyslipidemia were 63.2% and 78.9%, respectively. In addition, there was no change in the dose of any drugs for 6 months after the switching including the dose of 6 insulin users. Regarding side effects, gastrointestinal symptoms appeared in 4 cases in the early stage of switching, but they did not lead to discontinuation of drugs.

#### 3.1.2. Study 2 (Propensity Score Matching)

We set the control group who were continuously treated with dulaglutide without any changing in antidiabetic drugs during the same period in this study. The number of patients was 100. Secondly, to minimize the possible imbalance in the clinical parameters between each group at the baseline, we used 1 : 1 propensity score matching including 5 predefined baseline covariates as follows: age, gender, duration of diabetes, HbA1c, and BMI. Finally, each 13 cases were matched 1 : 1.

The primary endpoint in “study 2” was the difference in HbA1c change between the two groups. And, secondary endpoints included the difference in BMI change between the two groups and the difference in absolute HbA1c after 6 months. The clinical characteristic at baseline was shown in Table 1(b). The percentages of concomitant drugs in the nonswitching group and the switching group were as follows: DPP-4 inhibitor (0% vs. 0%), metformin (69.2% vs. 84.6%), thiazolidine (15.4% vs. 38.5%), sulfonylurea (15.4% vs. 23.1%), glinide (15.4% vs. 7.7%), *α*-glucosidase inhibitor (15.4% vs. 7.7%), SGLT2 inhibitor (46.2% vs. 76.9%), and insulin preparation (23.1% vs. 38.5%). There were no significant differences in the usage of concomitant drugs between the 2 groups.

### 3.2. Evaluation of Glycemic Control and Other Parameters after Switching from Once-Weekly GLP-1RA Dulaglutide to Semaglutide in Subjects with T2DM

#### 3.2.1. Study 1

The starting dose of semaglutide after switching was 0.25 mg (84.2%) and 0.5 mg (15.8%), respectively. Three months later, semaglutide dose was 0.25 mg (15.8%), 0.5 mg (73.7%), and 1.0 mg (10.5%), respectively. Six months later, the dose was 0.25 mg (0%), 0.5 mg (52.6%), and 1.0 mg (47.4%), respectively (Table 2(a)). Namely, before switching, all patients used 0.75 mg of dulaglutide, and after switching, about half of them used 0.5 mg of semaglutide during the 6-month observation period and the other half of them reached 1.0 mg.  Table 3(a) shows the parameters 3 months before switching, at baseline, and 3 and 6 months after switching. No significant difference was observed in each value during the 3 months before switching and at baseline. BMI significantly decreased 3 and 6 months after switching compared to baseline (from 30.4 ± 5.2 to 30.0 ± 5.4 and 30.0 ± 5.5 kg/m^2^). HbA1c values also significantly reduced 3 and 6 months after switching (from 8.2 ± 1.1 to 7.9 ± 0.9 and 7.6 ± 0.9%). Improved trend was observed in PG from baseline to 3 and 6 months later (from 156.9 ± 59.7 to 144.0 ± 34.8 and 143.7 ± 33.5 mg/dL). There was no difference in lipid profiles such as TG, HDL-C, and LDL-C between at baseline and 6 months later (TG: from 142.1 ± 65.6 to 167.6 ± 119.4 mg/dL, HDL-C: from 45.6 ± 9.4 to 47.9 ± 13.1 mg/dL, and LDL-C: from 94.2 ± 31.9 to 90.2 ± 23.3 mg/dL). ALT and *γ*-GTP levels were slightly decreased from baseline to after 6 months (ALT: from 33.4 ± 20.6 to 29.9 ± 14.7 U/L; *γ*-GTP: 32.6 ± 22.2 to 30.1 ± 26.2 U/L). Interestingly, AST level significantly lowered 6 months later (AST: from 23.7 ± 7.5 to 21.5 ± 5.7 U/L). There was no difference in serum creatinine, BUN, and eGFR levels between at baseline and 6 months later (creatinine: from 0.9 ± 0.4 to 0.9 ± 0.4 mg/dL, BUN: from 16.7 ± 6.4 to 16.5 ± 6.1 mg/dL, and eGFR: from 79.4 ± 25.2 to 79.4 ± 26.9 mL/min/1.73 m2). To examine which factors are involved in the effects of the change from dulaglutide to semaglutide on glycemic control, we estimated a possible association between *Δ*HbA1c (after 6 months-at baseline) and various clinical parameters at baseline (Table 4(a)). As the results, age, duration, BMI, hepatic enzymes, and lipid parameters were not associated with *Δ*HbA1c. Interestingly, however, the higher baseline HbA1c level was, the larger effect was obtained in *Δ*HbA1c after 6 months (*ρ* = −0.688, *p* < 0.001). To further examine which factors are involved in the effects of the change from dulaglutide to semaglutide on glycemic control, we divided the subjects into two groups: an improved group with ΔHbA1c < 0 and a nonimproved group with HbA1c ≥ 0. There was no difference in various parameters at baseline between the 2 groups except for HbA1c value (data not shown). Interestingly, however, HbA1c level at baseline was significantly higher in the improved group compared to the nonimproved group (HbA1c: 8.6 ± 1.0 vs. 7.2 ± 0.7%, *p* = 0.008). These data suggest that switching dulaglutide to semaglutide would bring about more favorable effects in clinical practice, especially in obese subjects with poorly glycemic control.

### 3.3. Comparison of Other Clinical Parameters between the Switching Group and the Dulaglutide Continuation Group

#### 3.3.1. Study 2 (Propensity Score Matching)

After the propensity score matching, at baseline, all parameters showed the same level between the 2 groups (Table 1(b)).  Table 2(b) shows the use of each drug in the 2 groups. As shown in Table 3(b), most values were not significantly different between baseline and 3 months before baseline in each group. This suggested that the reason for the switching was not due to the acute deterioration of glycemic control. In the dulaglutide continuation group, there were no significant changes in all parameters compared to that at baseline except LDL-C. In contrast, in the switching group, HbA1c was improved 6 months after switching from dulaglutide as well as BMI. There were no changes in lipid profile (TG, HDL-C, and LDL-C) and liver enzymes (ALT, AST). There were no significant differences between the dulaglutide continuation group and the switching group at each time point. We performed Spearman's rank correlation coefficient test for evaluating the relationship between *Δ*HbA1c (6 months-baseline) and baseline parameters (Table 4(b)). The negative correlation between *Δ*HbA1c and baseline HbA1c was revealed (*ρ* = −0.666, *p* = 0.013). We compared the changes for 6 months between the dulaglutide continuation group and the switching group (Table 5). There was a significant difference in changes in HbA1c and BMI (*p* = 0.049 and *p* = 0.042, respectively). Taken together, usage of once-weekly GLP-1RA semaglutide instead of the same class dulaglutide ameliorated glycemic control and reduced body weight in obese subjects with T2DM.

## 4. Discussion

In this study, we showed that switching once-weekly GLP-1RA dulaglutide to semaglutide significantly ameliorated glycemic control and reduced body weight in subjects with T2DM. In addition, the higher HbA1c was at the switching, the greater reduction was obtained in HbA1c value 6 months later. While semaglutide seemed to have stronger effects on glycemic and weight management than dulaglutide, the SUSTAIN 7 trial directly compared the effects of semaglutide and dulaglutide [14]. As the results, semaglutide had a more favorable effect than dulaglutide on glycemic control and weight management. However, this study was conducted in Europe and the United States and did not include Japanese patients. It is well known that East Asians and Westerners have different pathological conditions in subjects with T2DM. In Japanese, insulin secretary capacity is much smaller than that of Westerners; that is, the main cause of T2DM is impaired insulin secretion, but not insulin resistance [16, 17]. In addition, 1.5 mg of dulaglutide, which is not approved in Japan, was used in SUSTAIN 7. From these points, it seems inappropriate to immediately apply the result to SUSTAIN 7 into Japanese patients with T2DM. On the other hand, in a study in which 0.75 mg of dulaglutide was administered to Japanese patients with T2DM for 26 weeks, HbA1c value was reduced by 1.43%, but body weight was decreased by only 0.02 kg [18]. Similarly, in a semaglutide study in Japanese subjects with T2DM, the mean HbA1c value was reduced by 1.9% and 2.2% with 0.5 mg and 1.0 mg of semaglutide after 30 weeks, respectively. Body weight was decreased by 2.2 kg and 3.9 kg with 0.5 mg and 1.0 mg of semaglutide, respectively [19]. From these two trials, semaglutide seems to have an advantage, but differences in patient background and trial design need to be considered. As a good example, in various trials for evaluating cardiovascular outcome, the effect of reducing cardiovascular event risk was initially observed only with the human GLP-1 analog preparation, but not the GLP-1 preparation derived from exendin-4 [20]. Therefore, there was a tendency to think that antiarteriosclerotic effect was a drug effect of human GLP-1 analog. However, since it was reported in 2021 that efpeglenatide derived from exendin-4 suppressed cardiovascular events during a two-year observation period [21], this effect can be considered as a class effect of GLP-1 preparation. Considering these points, the influence of differences in patient background and study design on the results is not small, and caution is required in interpreting the results of clinical studies. Furthermore, there have been a few reports showing possible effects of switching from one GLP-1RA to another one on glycemic control and weight management. A similar study was conducted in a prospective study [22]. Switching from dulaglutide to semaglutide improved not only HbA1c value but also body weight and metabolic parameters, which was consistent with our results. However, the psychological effects of switching may be involved in the results, and although this is a retrospective study, in our study, we believe that establishing a nonswitching group using propensity score matching is of considerable significance. In this study, we showed that body weight was significantly decreased after switching to semaglutide even in background-matched analyses. We think that one possible underlying mechanism for this is due to the lower molecular weight of semaglutide compared to dulaglutide (4 kDa vs. 63 kDa), which facilitates its uptake into the brain through the blood-brain barrier (BBB) and thus leads to appetite and weight loss [23]. In general, the use of GLP-1RA with a higher molecular weight has a lower risk of gastrointestinal symptoms compared to GLP-1RA with a lower molecular weight. Indeed, the effects of GLP-1RA on the central nervous system (CNS) have not been fully elucidated. Hindbrain regions located outside the BBB are key input sites for vomiting and nausea responses [24]. Circulating GLP-1RA can act directly on this region without crossing the BBB. On the other hand, GLP-1RA, which can cross the BBB, has a greater impact on the CNS and a higher risk of nausea. Semaglutide, which has a smaller molecular weight, crosses the BBB and acts in a specific area, presumably resulting in a stronger weight loss effect. In addition, since GLP-1 receptor level is reduced under diabetic conditions, earlier use of GLP-1RA is thought to have stronger effect in view of *β*-cell protection. In fact, we reported that GLP-1RA had a protective effect against *β*-cell dysfunction [25] and its effectiveness was more pronounced at an early stage of diabetes [26]. Moreover, it is generally known that after chronic exposure of some receptor to a high concentration of its ligand, its receptor expression level is downregulated [27]. Thereby, it seems that long-term treatment with GLP-1RA downregulates its receptor expression, because serum GLP-1 level is increased to nonphysiological concentration after treatment with GLP-1RA. However, we recently showed that the use of GLP-1RA dulaglutide for a long period of time did not reduce *β*-cell GLP-1 receptor expression levels, thus contributing to the maintenance of good glycemic management [28]. Of interest is a report from Italy that the effect of liraglutide is more effective in women [29]. On the other hand, it has been reported from Japan that dulaglutide has less variability to body weight fluctuations in women than in men [30]. These reports suggest a gender difference in the effects of GLP-1 receptor agonists. Unfortunately, due to the small sample size, this study was not able to examine this point.

Based on all findings in this clinical study, we interpret the current data from the clinical point of view as follows. First, once-weekly GLP-1RA semaglutide had more favorable effects on glycemic control and weight management in subjects with type 2 diabetes mellitus in this study. Therefore, we think that when we fail to obtain good glycemic control and/or weight management with dulaglutide, it would be better to switch the medication from dulaglutide to semaglutide. Second, the above-mentioned switching showed more favorable effects on glycemic control especially in obese subjects in this study. Therefore, we think that when we fail to obtain good glycemic control with dulaglutide in obese subjects, we should switch the medication from dulaglutide to semaglutide. Third, there was no association between the effects of the switching and age or diabetes duration in this study. Therefore, we think that it would be better to switch the medication irrespective of age and diabetes duration. It is known, however, that semaglutide has more side effects such as gastrointestinal symptoms compared to dulaglutide especially in elderly subjects [31, 32]. Therefore, we should bear this point in mind as well in clinical practice.

This study has some limitations. First, the number of subjects was small to conclude the usefulness of semaglutide rather than dulaglutide. The number of subjects in study 1 (single arm) was 19, and the number of subjects in study 2 (after matching) was 13. Unfortunately, we were unable to obtain sufficient urinary microalbumin data to withstand the analysis. Furthermore, although PS matching established a control group with matching backgrounds as much as possible, the psychological impact of switching on patients cannot be ruled out. Although our current study suggested that the usage of semaglutide instead of dulaglutide would be useful in obtaining better glycemic and weight management in obese subjects with T2DM, further prospective clinical trials with a larger number of subjects would be needed to distinguish the effectiveness and characteristics between dulaglutide and semaglutide. In addition, although such effects were obtained irrespective of age, body weight, and diabetes duration in this study, further studies should clarify in more detail as to make the most of the potential effects of semaglutide in clinical practice.

In conclusion, at least in obese subjects with poor glycemic control, switching once-weekly GLP-1RA dulaglutide to semaglutide showed more favorable effects on glycemic control and weight management irrespective of age, body weight, and diabetes duration. Therefore, we should bear in mind that it would be better to start using a relatively new once-weekly GLP-1RA semaglutide in clinical practice, especially in obese subjects with poor glycemic control with other GLP-1RAs.

## Figures and Tables

**(a):** 

Parameter	Mean ± SD	Parameter	Mean ± SD
Number	19	HDL-C (mg/dL)	45.6 ± 9.4
Gender (M/F)	15/4	LDL-C (mg/dL)	94.2 ± 31.9
Age (years)	53.1 ± 11.1	*γ*-GTP (U/L)	32.6 ± 22.2
Duration (years)	16.9 ± 10.4	ALT (U/L)	33.4 ± 20.6
HbA1c (%)	8.2 ± 1.1	AST (U/L)	23.7 ± 7.5
PG (mg/dL)	156.9 ± 59.7	Cre (mg/dL)	0.9 ± 0.4
BMI (kg/m^2^)	30.4 ± 5.2	BUN (mg/dL)	16.7 ± 6.4
TG (mg/dL)	142.1 ± 65.6	eGFR (mL/min/1.73m^2^)	79.4 ± 25.2

**(b):** 

Parameter	Dula continuation	Switching	*p* value
Age (years)	47.5 ± 11.8	54.0 ± 9.9	0.200
Gender (M/F)	8/5	10/3	0.394
Duration (years)	14.8 ± 9.7	16.5 ± 10.9	0.878
BMI (kg/m^2^)	29.1 ± 6.7	29.2 ± 3.0	0.798
HbA1c (%)	7.4 ± 1.6	8.1 ± 1.1	0.143
PG (mg/dL)	161.8 ± 111.8	161.0 ± 69.9	0.555
TG (mg/dL)	132.9 ± 67.4	144.5 ± 73.7	0.644
HDL-C (mg/dL)	43.5 ± 11.6	43.4 ± 8.6	0.764
LDL-C (mg/dL)	82.6 ± 24.4	90.1 ± 27.1	0.521
*γ*-GTP (U/L)	32.7 ± 34.9	35.5 ± 24.2	0.175
ALT (U/L)	27.7 ± 13.1	34.2 ± 21.7	0.258
AST (U/L)	22.1 ± 9.7	24.7 ± 7.6	0.292
Cre (mg/dL)	0.9 ± 0.3	0.9 ± 0.4	0.356
BUN (mg/dL)	22.1 ± 19.1	16.2 ± 6.6	0.478
eGFR (mg/dL)	62.8 ± 24.1	74.3 ± 22.1	0.166

Abbreviations: BMI: body mass index; PG: plasma glucose; TG: triglyceride; HDL-C: high-density lipoprotein cholesterol; LDL-C: low-density lipoprotein cholesterol; *γ*-GTP: *γ*-glutamyl transpeptidase; ALT: alanine aminotransferase; AST: aspartate aminotransferase; Cre: creatinine; BUN: blood urea nitrogen; eGFR: estimated glomerular filtration rate. Data are shown as mean ± SD.

**(a):** 

Dose	At baseline, *n* (%)	After 3 months, *n* (%)	After 6 months, *n* (%)
0.25 mg	16 (84%)	3 (16%)	0 (0%)
0.5 mg	3 (16%)	14 (74%)	10 (53%)
1.0 mg	0 (0%)	2 (11%)	9 (47%)
Average dose	0.29 ± 0.09	0.51 ± 0.19	0.74 ± 0.26

**(b):** 

Dose		At baseline, *n* (%)	After 3 months, *n* (%)	After 6 months, *n* (%)
Dula	0.75 mg	13 (100%)	13 (100%)	13 (100%)
	0.25 mg	11 (85%)	3 (23%)	0 (0%)
Sema	0.5 mg	2 (15%)	8 (62%)	7 (54%)
	1.0 mg	0 (0%)	2 (15%)	6 (46%)
Average dose		0.29 ± 0.09	0.52 ± 0.24	0.73 ± 0.26

**(a):** 

Parameter	-3 months	Baseline	3 months	6 months
BMI (kg/m^2^)	30.5 ± 5.2	30.4 ± 5.2	30.0 ± 5.4^∗^	30.0 ± 5.5^∗^
HbA1c (%)	8.1 ± 1.3	8.2 ± 1.1	7.9 ± 0.9^∗^	7.6 ± 0.9^∗^
PG (mg/dL)	160.4 ± 71.0	156.9 ± 59.7	144.0 ± 34.8	143.7 ± 33.5
TG (mg/dL)	142.7 ± 73.6	142.1 ± 65.6	150.9 ± 65.6	167.6 ± 119.4
HDL-C (mg/dL)	44.7 ± 8.9	45.6 ± 9.4	46.6 ± 11.0	47.9 ± 13.1
LDL-C (mg/dL)	90.9 ± 29.6	94.2 ± 31.9	93.4 ± 30.3	90.2 ± 23.3
*γ*-GTP (U/L)	31.5 ± 21.0	32.6 ± 22.2	33.4 ± 32.9	30.1 ± 26.2
ALT (U/L)	32.8 ± 17.1	33.4 ± 20.6	33.1 ± 18.8	29.9 ± 14.7
AST (U/L)	23.0 ± 6.1	23.7 ± 7.5	23.3 ± 7.0	21.5 ± 5.7^∗^
Cre (mg/dL)	0.9 ± 0.3	0.9 ± 0.4	0.9 ± 0.4	0.9 ± 0.4
BUN (mg/dL)	16.9 ± 5.2	16.7 ± 6.4	17.2 ± 6.2	16.5 ± 6.1
eGFR (mL/min/1.73m^2^)	81.3 ± 25.2	79.4 ± 25.2	78.9 ± 26.3	79.4 ± 26.9

**(b):** 

Parameter	Dula continuation				Switching			
	-3 months	Baseline	3 months	6 months	-3 months	Baseline	3 months	6 months
BMI (kg/m^2^)	30.2 ± 6.9^∗^	29.1 ± 6.7	29.0 ± 7.2	29.3 ± 7.6	29.3 ± 3.0	29.2 ± 3.0	28.8 ± 3.0^∗^	28.7 ± 3.0^∗^
HbA1c (%)	7.6 ± 1.5	7.4 ± 1.6	7.6 ± 2.3	7.4 ± 1.7	7.9 ± 1.0	8.1 ± 1.1	7.8 ± 1.0	7.4 ± 0.8^∗^
PG (mg/dL)	162.4 ± 72.0	161.8 ± 111.8	163.6 ± 110.8	146.2 ± 76.3	152.2 ± 53.6	161.0 ± 69.9	143.1 ± 40.4	137.5 ± 34.4
TG (mg/dL)	155.4 ± 89.5	132.9 ± 67.4	153.2 ± 98.5	111.0 ± 30.8	133.5 ± 65.1	144.5 ± 73.7	148.1 ± 64.0	176.0 ± 137.4
HDL-C (mg/dL)	43.5 ± 12.4	43.5 ± 11.6	44.8 ± 16.8	47.5 ± 16.5	42.5 ± 6.3	43.4 ± 8.6	45.1 ± 9.0	45.7 ± 10.9
LDL-C (mg/dL)	82.5 ± 27.0	82.6 ± 24.4	82.1 ± 26.9	91.5 ± 29.8^∗^	89.8 ± 30.0	90.1 ± 27.1	94.8 ± 28.7	88.8 ± 25.4
*γ*-GTP (U/L)	31.4 ± 24.4	32.8 ± 34.9	35.9 ± 35.0	35.2 ± 37.3	33.9 ± 23.4	35.5 ± 24.2	36.9 ± 38.4	34.5 ± 30.2
ALT (U/L)	23.2 ± 7.6	27.7 ± 13.1	26.5 ± 13.3	25.2 ± 14.1	32.3 ± 15.0	34.2 ± 21.7	32.3 ± 15.7	30.8 ± 14.5
AST (U/L)	20.1 ± 5.1	22.1 ± 9.7	23.5 ± 9.5	24.2 ± 17.9	23.3 ± 5.2	24.7 ± 7.6	23.8 ± 5.4	22.4 ± 5.0
Cre (mg/dL)	0.9 ± 0.4	0.9 ± 0.3	0.9 ± 0.4	0.9 ± 0.4	0.9 ± 0.3	0.9 ± 0.4	0.9 ± 0.4	0.9 ± 0.5
BUN (mg/dL)	20.2 ± 15.7	22.1 ± 19.1	19.1 ± 11.9	18.2 ± 8.4	17.3 ± 5.3	16.2 ± 6.6	17.1 ± 7.0	17.0 ± 7.0
eGFR (mL/min/1.73m^2^)	67.7 ± 27.0	62.8 ± 24.1	67.5 ± 25.9	62.2 ± 24.2	76.4 ± 21.0	74.3 ± 22.1	73.5 ± 21.3	74.0 ± 24.1

Abbreviations: BMI: body mass index; PG: plasma glucose; TG: triglyceride; HDL-C: high-density lipoprotein cholesterol; LDL-C: low-density lipoprotein cholesterol; *γ*-GTP: *γ*-glutamyl transpeptidase; ALT: alanine aminotransferase; AST: aspartate aminotransferase; Cre: creatinine; BUN: blood urea nitrogen; eGFR: estimated glomerular filtration rate. Data are shown as mean ± SD. ^∗^*p* < 0.05 vs. baseline.

**(a):** 

Univariate analysis		
	*Δ*HbA1c	
Parameter	*ρ*	*p*
Age (years)	0.023	0.927
Duration (years)	-0.064	0.795
HbA1c (%)	-0.688	<0.001
PG (mg/dL)	-0.146	0.551
BMI (kg/m^2^)	0.038	0.880
*γ*-GTP (U/L)	0.247	0.323
ALT (U/L)	0.328	0.171
AST (U/L)	0.391	0.100
TG (mg/dL)	-0.392	0.107
HDL-C (mg/dL)	0.096	0.705
LDL-C (mg/dL)	-0.396	0.093
eGFR (mL/min/1.73m^2^)	-0.218	0.370

**(b):** 

Univariate analysis (switching)		
	*Δ*HbA1c	
Parameter	*ρ*	*p*
Age (years)	0.204	0.503
Duration (years)	0.040	0.897
HbA1c (%)	-0.666	0.013
PG (mg/dL)	-0.237	0.436
BMI (kg/m^2^)	0.253	0.404
*γ*-GTP (U/L)	0.306	0.360
ALT (U/L)	0.199	0.515
AST (U/L)	0.506	0.078
TG (mg/dL)	-0.263	0.409
HDL-C (mg/dL)	0.246	0.442
LDL-C (mg/dL)	-0.281	0.353
eGFR (mL/min/1.73m^2^)	-0.237	0.436

Abbreviations: BMI: body mass index; PG: plasma glucose; TG: triglyceride; HDL-C: high-density lipoprotein cholesterol; LDL-C: low-density lipoprotein cholesterol; eGFR: estimated glomerular filtration rate.

**Table 5:** Comparison of changes in HbA1c and BMI for 6 months between the dula continuation group and the switching group. Study 2.

Parameter	Dula continuation	Switching	*p* value
*Δ*HbA1c (%)	−0.015 ± 0.508	−0.615 ± 0.832	0.048
*Δ* BMI (kg/m^2^)	0.267 ± 1.096	−0.470 ± 0.553	0.042

Abbreviations: BMI: body mass index. Data are shown as mean ± SD.

## Data Availability

The data that support the findings of this study are not publicly available due to privacy or ethical restrictions.

## Abbreviations

BMI: Body mass index
PG: Plasma glucose
TG: Triglyceride
HDL-C: High-density lipoprotein cholesterol
LDL-C: Low-density lipoprotein cholesterol
*γ*-GTP: *γ*-Glutamyl transpeptidase
ALT: Alanine aminotransferase
AST: Aspartate aminotransferase
Cre: Creatinine
BUN: Blood urea nitrogen
UA: Uric acid
eGFR: Estimated glomerular filtration rate.
